# Medicines, Their Use and Mode of Administration

**Published:** 1845-09

**Authors:** 


***Medicines, their Uses and Mode of Administration; including a complete Conspectus of the three British Pharmacopœias, an Account of all the New Remedies, and an Appendix of Formulæ.*** By J. MOORE NELIGAN, M. D., Physician to Lewis street Hospital, and Lecturer on Materia Medica and Therapeutics in the Dublin School of Medicine. With Notes and Additions, conforming it to the Pharmacopœia of the United States, and including all that is new or important in recent improvements. By DAVID MEREDITH REESE, A. M., M. D., Late Professor of the Institutes of Medicine and Surgery in the Washington University of Baltimore, &c. pp. 453, 8vo. New York: HARPER & BROTHERS, 1844.

The above-named work commends itself to the student of medicine and medical practitioner, not only for its brevity, but also for the very concise, and, at the same time, complete account which it contains of the different medicinal substances, both simple and compound, which have place in the materia medica, as well as that of those of more recent discovery.

Avoiding the usual natural-historical or alphabetical arrangement, the author adopts a physiological classification, "based on the ultimate effects, of remediate agents."

"In describing each medicinal substance, the following plan is adopted:

"1st. The officinal appellation and English name of each article is given; and, in the case of vegetable substance, the native country and botanical classification of the plant from which it is obtained. For the advantage of the student, the most important characters of each medicinal plant are also concisely described.

"2d. The physical properties.

"3d. The chemical properties.

"4th. The mode of preparation. Under this head, the process of the three British pharmacopœias are given in full.

"5th. The adulterations, and the manner in which they may be detected.

"6th. The therapeutical effects, and the uses of the substance in the treatment of disease.

"7th. The dose and mode of administration. Under this head, all the officinal preparations of the British pharmacopœias, as well as many of those ordered by the continental and American colleges, are introduced.

"8th. The incompatibles.

"9th. In the case of poisons, the antidote and mode of treatment."

We regard this as a work of peculiar merit, and its value is greatly enhanced by the labors of the learned American editor, in conforming the nomenclature and pharmaceutics of the volume to the pharmacopœia of the United States. Dr. Reese has also supplied a "number of new remedies, and many officinal preparations, both old and new," which were omitted by the author. In addition to which, there are, interspersed throughout the work, valuable critical and practical notes, from the pen of this able writer and eminent physician.

To the medical profession, we doubt not that this work is already extensively and favorably known. Our object, in noticing it at this time, is to call the attention of our own immediate professional brethren to it.

